# Family Planning Knowledge, Attitudes, and Practices among Married Men and Women in Rural Areas of Pakistan: Findings from a Qualitative Need Assessment Study

**DOI:** 10.1155/2015/190520

**Published:** 2015-09-01

**Authors:** Ghulam Mustafa, Syed Khurram Azmat, Waqas Hameed, Safdar Ali, Muhammad Ishaque, Wajahat Hussain, Aftab Ahmed, Erik Munroe

**Affiliations:** ^1^Marie Stopes Society, Research, Monitoring and Evaluation, Technical Services Department, 21-C, Commercial Area, Old Sunset Boulevard, DHA-II, Karachi, Sindh 75500, Pakistan; ^2^Department of Urogynecology, Faculty of Medicine and Health Sciences, University of Gent, Sint-Pietersnieuwstraat 25, 9000 Gent, Belgium; ^3^Marie Stopes International, Research, Monitoring and Evaluation Department, 1 Conway Street, Fitzroy Square, London W1T 6LP, UK

## Abstract

This paper presents the findings of a qualitative assessment aimed at exploring knowledge, attitudes, and practices regarding family planning and factors that influence the need for and use of modern contraceptives. A descriptive exploratory study was conducted with married women and men aged between 15 and 40. Overall, 24 focus group discussions were conducted with male and female participants in three provinces of Pakistan. The findings reveal that the majority knew about some modern contraceptive methods, but the overall contraceptive use was very low. Knowledge and use of any contraceptive method were particularly low. Reasons for not using family planning and modern contraception included incomplete family size, negative perceptions, in-laws' disapproval, religious concerns, side-effects, and lack of access to quality services. The majority preferred private facilities over the government health facilities as the later were cited as derided. The study concluded the need for qualified female healthcare providers, especially for long term family planning services at health facilities instead of camps arranged occasionally. Addressing issues around access, affordability, availability, and sociocultural barriers about modern contraception as well as involving men will help to meet the needs and ensure that the women and couples fulfill their childbearing and reproductive health goals.

## 1. Background

Despite being the sixth most populous country on the planet with the population exceeding 184 million, Pakistan is facing a huge challenge of poverty where 61% of its population is living below US$2 a day [1, 2]. About 45% of its population has limited access to health services both public and private, especially in rural areas where 65% of its population resides [2]. The country lags far behind on almost all development indicators, particularly with regard to maternal and child health [3]. It has been estimated that approximately 28,000 women die annually in Pakistan due to preventable pregnancy-related complications [2]. In 2008, Pakistan was included amongst the six countries that contributed to more than 50% of maternal deaths happening worldwide [3].

Maternal and neonatal health are strongly interlinked. Around 33% of neonates in Pakistan die due to maternal infections and other problems related to pregnancy and delivery [4]. The level of health among Pakistani women is alarmingly poor and contributes to both maternal and child morbidity and mortality. Some estimates from recent studies suggest that the lifetime risk of maternal death for Pakistani women is one in 93 [5]. Only half of the deliveries in Pakistan take place in the presence of skilled health provider and rural and less educated women are less likely to revive skilled delivery care [2]. Not only maternal mortality is high in Pakistan but there is a substantial rural-urban differential in maternal mortality (319 versus 175 per 100,000, resp.) [2]. Antenatal care coverage is far from optimal; 27% of pregnant women in Pakistan still receive no antenatal care and 40% do not receive postnatal care after delivery [2].

In addition to maternal health, newborn health and survival are another priority area for improvement. The Pakistan Demographic and Health Survey (PDHS) 2006-7 indicates that the neonatal mortality rate (NMR) has remained virtually unchanged over the past 15 years. It highlighted regional disparities also in this regard [6]. For example, the 10-year NMR is much higher in Punjab (58 per 1,000) and Sindh (53 per 1,000) than in NWFP and Baluchistan (41 and 30 per 1,000, resp.).

Modern FP methods, which have been documented to be highly effective means of improving maternal health by preventing unintended pregnancies in order to ensure healthy timing and spacing of births, only account for 26% of FP use in Pakistan. Moreover, the overall levels of FP use in rural areas continue to remain very low (around 31%), compared to 45% in urban areas. Similarly, women from the poorest households and those with no education have the lowest CPR [2].

Furthermore, the PDHS 2012-13 documents a significant unmet need for contraception at 20% [2]. According to an estimate, 890,000 induced abortions occur annually in Pakistan whereby one in seven pregnancies is terminated by induced abortion often performed in clandestine conditions [7] and abortion being used as means to control fertility and an outcome of failed contraception [7]. Out of the total fertility rate (TFR) of 3.8 in Pakistan, one birth is unwanted [2]. There are a number of structural and sociocultural issues that pose a challenge to improving maternal and newborn health (MNH) status in Pakistan. Lack of money, transportation, denial of family permission, or/and distance from health facility are some of the critical problems the majority of women face in Pakistan [2].

The average distance to a reproductive health facility in rural areas is larger than that to urban areas which makes access to services for rural women without transportation or funds extremely difficult [2]. It is also noteworthy that despite the large government infrastructure of primary, secondary, and tertiary care facilities in many areas throughout the country, as well as a Lady Health Worker (LHW) program, more than 70% of the population seeks healthcare through the private sector [6].

In addition, the dynamics of decision-making between a husband and wife also create barriers to access. Several studies have examined the influence of social and cultural factors on contraceptive use in Pakistan [8]. These studies have emphasized the influence of the mother-in-law and the husband on family planning decision-making [9, 10] and have highlighted the importance of communication between spouses regarding the use of contraception [11, 12]. Despite the huge benefits, family planning is one of the most difficult and least discussed topics, particularly amongst males in a conservative and patriarchal society where men have the final decision-making power regarding most issues, including reproductive health. Nevertheless, there have been some efforts to target men through either advocacy or behavioral change interventions, but very little have been achieved.

Healthy timing and spacing of pregnancy (HTSP) is a family planning intervention to help women and couples delay, time, space, or limit their pregnancies to achieve the healthiest outcomes for women, newborns, infants, and children regardless of the total number of children [13]. It has been documented that perinatal outcomes and child survival can be improved mainly by lengthening interpregnancy intervals [14, 15]. Over one million maternal deaths were averted between 1990 and 2005 because the fertility rate in developing countries has declined and by reducing high parity births family planning contributed to reducing the maternal mortality ratio [16]. On the contrary, birth to pregnancy intervals of less than 18 months are associated with risk of low birth weight, preterm birth, small size for gestational age, and stillbirth [17]. Despite the increased awareness and acknowledgement of birth spacing in improving maternal and child health outcomes, there is little evidence on effective, scalable, and sustainable programs for birth spacing in developing countries like Pakistan, particularly in rural areas. Moreover, there is a need to package evidence in creative ways to support program and policy decision-making at multiple levels: from community to policy arenas of Pakistan [18].

“Evidence for Innovating to Save Lives” was a 36-month operations' research initiative, implemented by Marie Stopes Society (MSS) from 2010 to 2013 aiming to promote birth spacing and modern contraceptive uptake in poor segments of population of Pakistan (http://mariestopespk.org/raf/research-project/). The overall goal of the project was to improve MNH through healthy timing and spacing of pregnancies in rural and underserved areas of Sindh, Punjab, and Khyber Pakhtunkhwa (KPK) provinces of Pakistan. Before the implementation of the project, MSS conducted a qualitative need assessment study with the potential target population, including men and women, in proposed project areas. The study aimed to explore knowledge, attitudes, and practices concerning family planning and birth spacing; perceptions about quality of care; health seeking behavior; community need assessment; and barriers and facilitators that influence contraceptive uptake. This paper presents the findings of the need assessment study.

## 2. Study Design and Methodology

Using the descriptive exploratory design, the need assessment study employed Focus Group Discussions (FGDs) technique for data collection. Overall, twenty-four FGDs were conducted, 8 FGDs with males and 16 FGDs with females. The FGDs took place in 2 districts of Sindh, 3 districts of Punjab, and 3 districts of Khyber Pakhtunkhwa provinces of Pakistan (refer to Table 1).

### 2.1. Inclusion/Exclusion Criteria and Recruitment

The participants for the FGDs were selected randomly from the household who were fathers and mothers having at least one child less than 2 years and no child above 2 years; having total household income of about 6000 Rupees per month (US 60$); and residing in houses classified as Kacha (made of clay) or Semi-Pakka (made of clay and some concrete). Using a specially designed screening/recruitment questionnaire which was made in line with the preset criteria of the target participants, the researchers visited households in the villages in person and identified the potential participants and recruited the eligible participants for the FGDs with their informed consent. The households were selected on the basis of their placement within the 2-3-kilometer catchment areas of the nearest reproductive health services delivery outlet. Local community health workers, from public and private sectors, contributed to the identification of potential households and only the ones meeting the set criteria (all as mentioned above) were invited to participate in the FGDs.

### 2.2. Target Population and Sampling

The study targeted young and newly married men and women with no child older than two years as this is the reproductive age group mainly identified in Pakistan Demographic Health Survey (DHS) 2006-7 and 2012-13 lacking contraceptive exposure [2, 6]. The participants of FGDs were in an age group of 18–40 years (male) and 15–30 years (female); refer to Table 1. All the participants had at least one child less than 2 years of age. One FGD with males and two FGDs with females were conducted in each of the eight project districts (making a total of 8 groups for males and 16 for females); refer to Table 1 for details.

### 2.3. Study Sites

The study was carried out in 8 villages of the 8 rural districts of Sindh, Punjab, and Khyber Pakhtunkhwa provinces in Pakistan (refer to Table 1 for details). As the study was conducted in remote rural villages of the study districts, arrangements were made to conduct FGDs at places which had easy access to participants and where they felt comfortable, for example, autaq/baithak (community hall style places for male participants), schools, and/or homes for females.

### 2.4. Research Instrument and Data Collection

FGD guides were designed and developed by the MSS research team in English and were translated into the national language Urdu after pretesting in order to accommodate professional and cultural validation. The interviews were conducted in participants' native language and the data were collected by qualified researchers comprised of male and female moderators. The moderators facilitating the FGDs were university graduates trained and experienced in social science research techniques and were fluent in local languages. Additional training on family planning and reproductive health concepts and qualitative methods of data collection was also provided to the moderators. The FGDs were carried out separately with male and female participants using separate guidelines due to cultural and local area sensitivities. Each FGD group consisted of 6 to 8 participants. As a standard procedure, male moderators conducted FGDs with males, whereas female moderators conducted FGDs with female groups. Each FGD was audiotape-recorded and, in addition to audiotape recording, field and observational notes were also taken by research assistants.

### 2.5. Data Analysis

Audio-recording of the FGDs was transcribed word to word/verbatim and translated from Urdu/local language into English by the researcher fluent in both languages. These transcripts were used for detailed analysis. Using the thematic analysis approach, the researchers read and reread all of the transcripts several times to be familiar with the data and to identify predetermined and emerging themes from the data. Along with the manual analysis technique employed in the initial phase of data analysis, the data was also coded and thematically analyzed using QSR NVIVO 8 software for Windows. The codes were further refined, combined, and categorized to develop additional codes for a detailed analysis.

### 2.6. Ethical Considerations

The ethical approval for the project was provided by Program Oversight Committee (POC) of Research and Advocacy Fund (RAF) and National Bioethics Committee (NBC) of Pakistan (Ref. number 4-87/10/NBC-43/RDC/). Further, all of the study participants were briefed about the purpose of the study and their right to refuse to answer any question or withdraw from the FGDs at any time. They were informed that there was no “right” or “wrong” answer and they were requested to express their opinions and thoughts freely. They were informed that their names or any identification leading to them will be kept strictly confidential and that their names will not appear in any report or publication resulting from this study. They were further informed that the audio-recordings and hard copies of the transcripts will be kept under lock and key and subsequently destroyed in due time. Before the start of FGDs, verbal and informed consent was also taken from the study participants.

## 3. Findings

The following themes and subthemes (refer to Table 2) were identified and used for thematic analysis of data. The detailed findings will follow this table of themes.

### 3.1. Sociodemographic Characteristics and Reproductive History

The majority of the participants, including men and women, belonged to a very low socioeconomic status (SES) with average monthly income around 6000 PKR (1 USD$ = 89 PKR). Mostly, they were living in a large joint family setup, especially in Sindh and Khyber Pakhtunkhwa. Females from KPK said,* “we live together with our in laws. Including the mother-in-law, father in law, sister-in-law, brother-in-law, his wife and children; we are all around 15 people living together.”* The majority of male and female participants had entered into marriage at an early age, especially females at a very young age ranging between 15 and 24 years and males between 20 and 30 years.

### 3.2. Pregnancy-Related Problems

There were some complications in last pregnancy and miscarriages experienced by participants in all regions. A female from KPK said,* “my first pregnancy resulted in a miscarriage at 5th month; I lifted something heavy and got a miscarriage,”* while a female from Punjab shared a quite different reason for her miscarriage and said,* “I had lost a child after 6 months of pregnancy, my uterus was weak, the doctor advised spacing to strengthen the uterus and now I have a child after almost two years.”* Participants from Sindh having miscarriage were quite uncertain to find any reason. A female from Sindh said,* “I lost a child when I was 40 days pregnant, there was no reason. I was just sitting at home and miscarried.”*

However, many women in KPK and Sindh opted for induced abortion to end their pregnancies often in a potentially harmful way. In some cases women even have to rely on Dais (traditional birth attendants) or visit private hospitals outside their village for an abortion owing to poverty as they either do not want to have a female child or they do not want more children. A female from Sindh said,* “they go to a private hospital for this, everything can be done there, and they get themselves aborted and cleaned there in secrecy.”* Another female from Sindh said,* “yes, there are women who find it difficult to raise children, so they waste the fetus.”*

### 3.3. Health Service Seeking Behavior

#### 3.3.1. General Health Service Seeking Behavior

There was lack of basic health facilities in respective rural areas/villages of Sindh, Punjab, and Khyber Pakhtunkhwa. It was found that the majority of men and women prefer to go to private hospitals/clinics if they could afford owing to the perceived availability of various facilities. A male from Punjab said,* “Private hospitals have all kinds of facilities; they have every machine, ultrasound, blood tests and ambulance.”* The majority of men and women in those rural areas heavily relied on quacks for many healthcare needs which include dispensers and compounders in most cases, who were engaged in private practice in their respective villages. Despite the awareness that these were not qualified doctors, they visited them for lack of a choice available to them. A female from KPK said,* “there is only one doctor available in our area. He does not have an MBBS degree. He is a local practitioner.”*

#### 3.3.2. Health Service Seeking Behavior regarding Family Planning/Reproductive Health

Absence of FP/RH across the regions was prevailing in the same intensity. The majority of men and women have to travel outside in case of emergency which in a way affect the choice of availing FP methods. A male from Sindh said,* “there is no such facility in our village to provide services on birth-spacing or birth related complication. We have to go to the main city Sakrand which is 14–16 kilometers away from our village.”*

The majority of men and women in Sindh and Punjab were satisfied with the role of LHWs as they provide information and services at doorsteps with concerns over the IUCD procedure performed by them. A male from Sindh said,* “LHWs provide information and contraceptive methods. We listen to their advice carefully and trust them altogether. But we don't trust the operation that they do because it is done in the camp which is set up for only 2-3 days.”*

In contrast, majority of women in KPK were not satisfied as LHWs in their area barely provided services at the doorstep. In addition, LHWs charge their late visit at night in case of emergency and they provide pills and injections to their relatives and neighbors only. A female from KPK said,* “Lady Health Workers get medicines from the government and they distribute them among their relatives.”*

### 3.4. Knowledge of and Perceptions about Family Planning and Modern Contraception

The men and women across the regions were familiar with different family planning methods, especially modern contraception except vasectomy. Among traditional methods, the majority of participants had little knowledge and were indifferent toward breast feeding as a natural way to avoid pregnancy. A male from Sindh said,* “We have heard that some women breast feed their children and don't get pregnant whereas in some cases women breast feed their children and from the second month they start getting their menses.”*

The majority of participants across the regions were assured that FP is essential for the health of the mother and child and welfare of the family. A female from KPK said,* “Because there is one earner and many dependents, if we plan a family only then can you survive on one source of earning.”* A male from Sindh said,* “if there are fewer children then we can raise them properly; providing better facilities, food, education, etc. to them.”*

Moreover, it is interesting to note that indifferent approaches to FP/birth spacing still strongly prevail in rural areas of Pakistan. The majority of men in Sindh and KPK still seemed resistant to accept the use of FP/birth spacing and did not seem much in favor of family planning for financial and religious reasons. A male from Sindh said,* “if one has limited resources then only he should do FP, but once he is in a position to afford then he should put a stop to FP, this is the right approach, but those who go for tubal ligation that is not the right way which is against the religion.”* However, it is alarming to note that some married adolescent women in Sindh and Punjab were intending to use contraception only when they complete their family size having 5-6 children. This attitude was prevailing mainly among the young men and women of these regions (19–23 years of age). A female from Sindh said,* “We don't want to do family planning, we are very young, once we will have 5 to 6 children then only we will think, some females do want to have less children but males wants them to produce more children.”*

### 3.5. Sources of Knowledge regarding Family Planning and Modern Contraception

The majority of men and women identified word of mouth as the main and most reliable and immediate source of information across the region. A female from Sindh said,* “We live in one village, so if anybody finds out any information they share it with each other.”* In addition, women with previous experience of contraceptive use and television were mentioned as important sources of information. A female from Punjab said,* “we find out about pills and injection from TV.”* Another female from KPK said,* “We have heard about different contraceptive methods from people around us who had used these methods.”*

### 3.6. Current Contraceptive Use and Behavior

The majority of men and women across all regions were not using any family planning method mainly because they wanted more children, had negative perceptions about family planning, or had concerns about side-effects and due to lack of access to information and services. A female from Punjab said,* “My cousin did family planning and after that she couldn't have children. She used the method of IUCD for spacing between children. But suddenly both of her children died and after that she cannot get pregnant.”*

In contrast, there were only few who used modern methods as these ensured better health of the mother and child. A female from KPK said,* “We did plan and tried that we should not have another child because our first child was too young; therefore we wanted to have another child once our first child was grown enough; so we used condoms.”* Method-wise, condoms were mostly preferred by men. To quote a female from Punjab,* “The idea of using a condom was my husband's; he had asked the doctor and decided.”* The majority of women perceived IUCD and tubal ligation relatively safer compared to pills and injections. A female from Sindh said,* “my neighbor told me to have IUCD inserted for five years. It is safe and I can decide when to remove it whenever I want.”*

### 3.7. Decision-Making regarding Contraceptive Use

Discussions about decision-making regarding family planning and modern contraception yielded that husband and in-laws mainly influence how, when, and whether to practice family planning and use contraception. A female from Sindh said,* “my mother-in-law and father-in-law say that I should have as many children as I can.”*

Despite that, there did exist some mutual understanding and communication found between few spouses in some areas across the regions. A female from Punjab informed that* “It is a mutual decision; the husband tells us to take advice from the doctor because she has a better understanding.”*

### 3.8. Barriers towards Family Planning and Modern Contraception

Findings of the study confirmed few barriers identified in previous studies. Nevertheless, findings provided some new insight into the perception that impedes the use of family planning/birth spacing.

#### 3.8.1. Religious Barriers

The majority of men across the region do not practice FP/birth spacing owing to religious reasons. Quoting various religious injunctions and traditions, they discussed that bearing many children is advocated by Islam and is also beneficial for growth of Muslim Ummah (the Muslim brotherhood). A male from KPK said,* “we don't do family planning because of our religion; The Prophet (PBUH) had said that one should marry a woman whose family has more children.”* Similarly, a female from Punjab described it thus:* “The religion prohibits it because the prophet said that the more children a woman bears the more my Ummah (Muslim brotherhood) will grow.”*

#### 3.8.2. Lack of Knowledge and Fear of Side-Effects

Across all regions, the majority of participants, especially women, associated different types of side-effects with different contraceptive methods, for example, dangerous for fertility, black spots and hair growing on face, and impotency and lack of knowledge. A male from Punjab said,* “People do not practice family planning mainly because they do not know how, when and what to do use,”* whereas a female from Punjab said that* “People don't use methods because of fear; someone woman I know got herself injected, and for a whole month she bled and became weak as a result.”*

#### 3.8.3. Social Stigma and Pressure

It is important to note that the majority of young married males and females do not practice FP owing to various social stigmas. Social pressure to bear more children is identified as a barrier towards family planning as parents with more children are seen with more respect. A male from Sindh said,* “people here feel very shy telling anybody at home or anywhere else that he is going for family planning services with his wife. They either laugh at you or scorn you.”* A female from KPK said,* “People say if there are more children they will prove helpful in the future; and they will bring honor to the family name.”*

#### 3.8.4. Husband/In-Laws Disapproval

Females do not practice family planning without their husbands and mothers-in-law approval. This is the most pivotal restriction to cope within Pakistan which is at times linked with sociocultural and health issues. A female from Sindh said,* “My husband does not allow me; he wants me to keep having children; some men say that spacing leads to illness and if a woman keeps having children then she is healthy.”*

#### 3.8.5. Restrictions on Female Mobility

It is interesting to note that stringent restrictions on female mobility emerged as a major barrier across the regions. Females are not allowed to step out alone without the permission of their husbands or mothers-in-law. Women going alone even for medical help are thought to bring dishonor to the family. A female from KPK said that* “we cannot go freely or alone to get family planning services. We need permission from our husband or mother-in-law.”*

#### 3.8.6. Lack of Access and Affordability to FP/RH Services

There were persistent problems of accessibility, affordability, and unavailability of the doctors prevailing in the rural/village areas of Pakistan. A male from Punjab said that* “The men and women in this area want to practice family planning but it is not within their reach; it is hard for them to reach family planning centers in other cities which are far away from our residence.”* It is interesting to note that affordability issue was mainly highlighted by the females as they mentioned that their husbands were earning limited incomes and they were dependent on their husband's income. A female from Sindh said,* “My husband's salary is spent in running the expenses of the household, so we cannot spend money on these things. Also there is no Centre here and to go to Sakrand you need to arrange conveyance and that requires extra expenditure.”*

In addition, the majority of men and women highlighted the importance of having female doctor in their respective areas as they were not comfortable to discuss gynecological or reproductive health issues with the male doctors due to various sociocultural boundaries. A female from Punjab said that* “There is no female doctor here; we need one because currently we have to travel to the city to see female doctor. It is difficult to consult a male doctor on reproductive health issues.”*

## 4. Discussion

Almost all of the participants had entered into marriage very early with women entering into marriage relatively earlier than men, which is similar to national figures that report that on average women enter into marriage at the age of 18. Both men and women reported that the ideal number of children they would like to have in a family is four comprising boys and girls with greater emphasis on boys. In line with findings of the PDHS 2012-13, males were reported as wanting more children than females [2]. Spouse (husband) involvement appears to be a key factor in deciding to take a family planning method and it is also equally important with regard to the number of children a couple will have [16]. Joint decision-making (both spouses) is rarely seen with regard to the number of children or the taking-up of an FP method.

As shown by previous research evidence [6], men and women, depending on the ability to afford, preferred to go to private health facilities as these were perceived to have sufficient and responsive staff, were well equipped, and provided quality services. Government health facilities, most of the participants claimed, either were dysfunctional or lacked staff/services and were discriminatory in provision of services where prompt and quality services were only provided to well-off people.

Most of the participants had knowledge of at least one modern contraceptive method with condom being the most commonly known method which is also concurrent with the findings of Pakistan DHS 2012-13 [2]. However, in contrast to the present national data [2], long acting Intrauterine Device (IUD) was the most commonly known method after condom and female sterilization and was considered safer and having fewer side-effects by women in comparison to short term methods including injectable and pills. It is interesting to note that word of mouth is the most common source of information for both men and women in these rural communities which is also supported by previous research findings [19].

Both positive and negative perceptions about FP were recorded during the discussions. On the positive side, both male and female participants stated that increased awareness and financial pressure were the two main factors making couples keep their family sizes smaller. Besides, lack of awareness and sociocultural pressures (including in-laws' pressure on females and peers' on males) and shyness negatively affected perceptions about family planning and discouraged family planning uptake.

Regarding current or ever use of contraception, only a few participants, either male or female, reported positively. The dominant reason for not using contraceptives included desire for more children. Hence, being qualitative in nature, this study was not able to measure the real “unmet need for contraception”; instead the study tried to capture the perceived need for contraceptive services which seem to be very low in the target communities due to the desire of having more children expressed by the majority of the participants. Therefore, there is no real need for contraception. Likewise, the participants were mostly young and newly married and no participant had more than two children which led to such low perceived need for contraception. This is also concurrent with Pakistan DHS 2012-13 results which demonstrate that contraceptive use increases with age and the number of children and reaches optimal level when the couples have achieved their desired number of children [2]. However, the study findings also draw attention to other factors like lack of awareness about the range of family planning methods, absence of health facilities providing quality family planning services, inability to afford the quality services in remote cities, and sociocultural issues like peer-pressure, restrictions on female mobility, and in-laws' disapproval. These findings are also in agreement with previous research studies [6]. Fear of side-effects also emerged as an important impediment to contraceptive use which is also a recurrent theme in many studies conducted in developing countries including Pakistan, India, Bangladesh, and Ethiopia [19–22]. In addition, religious concerns were also cited by some participants as a reason for not using contraception which was also reported as important factor impeding contraceptive adoption by previous national DHS surveys [2, 6]. Some other studies have also highlighted religions as an important factor influencing an individual's decision to adopt contraception [23].

Nevertheless, the study findings need to be treated cautiously to avoid overgeneralization due to some limitations mainly arising from the study design. First of all, this is a qualitative study and is conducted with a limited number of people and may not represent the views of the whole population in the targeted communities. Secondly, the study documented the opinions of the married men and women who mostly were newly married, had no more than two children, and belonged to lower socioeconomic status and thus their opinions may vary from the opinions of men and women with different sociodemographic characteristics.

## 5. Conclusion and Recommendations

The study provides insights into the local contexts related to family planning knowledge, attitudes, perceptions, and practices and also highlights the need for contraceptives, especially for long acting and reversible contraceptives. In the wake of changing attitudes towards family planning and desired family size, more women and couples will be seeking family planning services. Addressing obstacles such as access, affordability, and availability will help meet these needs and ensure that women and couples can meet their childbearing and reproductive health goals. In addition, a very low perceived need for contraception was found amongst the respondents wanting more children expressed almost equally by male and female respondents.

The study also identified the need for qualified and trained female healthcare providers, especially for long term family planning services, including IUD, at well-established health facilities instead of camps setup occasionally. Both male and female participants were of the opinion that a female professional (ideally a doctor) should be made available in their areas to which people can have a quick and easy access. The men also emphasized that a male with training and adequate knowledge in family planning should be made available in their communities to inform men about the benefits of family planning and birth spacing.

In addition, the study findings reveal that mostly men and women do not use contraception either because they are newly married or because they have few children. Despite this, many young women and men expressed their intention to use contraception, though late in married life, depending on the quality and availability of the services. Well-targeted behavior change and communication campaigns can change the attitudes regarding birth spacing practices. These behavior change campaigns should encourage both men and women to adopt healthy birth spacing practices from the start or during the early period of marriage instead of letting them wait for completing their desired family size and then starting with contraception as is the current practice.

Moreover, the study also identified strong need for involving men in healthcare programs designed to improve women's and newborns' health as they mostly influence decision-making at the household level and this will also result in active male participation and community ownership. Young, especially first time, fathers need support and empowerment. Encouraging communication between wife and husband about family planning and birth spacing should also be part of such campaigns to promote mutual decision-making between wife and husband and make husbands responsible partners in family planning/birth spacing decisions and ease the burden of decision-making on women.

Furthermore, family planning and birth spacing interventions need to focus on alleviating fears about side-effects among men and women through effective counseling and providing adequate information to both men and women about method-related side-effects and how to manage them. In addition, involving community leaders, religious clerics, and health workers in awareness raising campaigns can help address sociocultural and religious concerns.

## Figures and Tables

**Table 1:** Area- and participant-wise data collection details of the study participants.

Province	District	Male		Female	
		Number of FGDs	Age group (in years)	Number of FGDs	Age group (in years)
Sindh	Nawabshah	1	18–24	2	(15–18) (19–23)
	Naushahro Feroze	1	26–39	2	(24–30) (15–18)

Lower Punjab	Bahawalpur	1	18–24	2	(19–23) (24–30)
	Khanewal	1	26–39	2	(15–18) (19–23)
	Pakpattan	1	18–24	2	(24–30) (15–18)

Khyber Pakhtunkhwa	Haripur	1	26–39	2	(19–23) (24–30)
	Mansehra	1	25–30	2	(18–24) (19–23)
	Abbottabad	1	26–39	2	(24–30) (18–24)

**Table 2:** Identified themes and subthemes.

Themes	Subthemes
Sociodemographic profile	Socioeconomic status
	Household structure
	Family size

Reproductive history	Approximate age at marriage
	Number of pregnancies and any history or experience of miscarriages or pregnancy termination
	Number of children
	Desired family size

Health seeking behavior	General health service seeking behavior
	Health service seeking behavior regarding family planning/reproductive health

Knowledge, attitudes, perceptions, and practices about family planning and modern contraceptive methods	Type of contraceptive methods known including modern contractive methods
	Perceptions about safety/effectiveness of contraceptive methods
	Knowledge, attitudes, perceptions, and practices regarding family planning and contraceptive methods

Sources of knowledge	Interpersonal including friends and relatives
	Mass media including radio, TV and cable, and newspaper

Current contraceptive practices	Modern contraceptive methods
	The preferred method
	Reasons of use, nonuse, and discontinuation

Decision-making regarding contraceptive use	Decision-making dynamics about family planning and spousal communication

Barriers on family planning and contraceptive use	Religious barriers
	Lack of knowledge
	Fear of side-effects
	Social stigma and social pressure
	Husband/in-laws' disapproval
	Lack of access
	Lack of affordability
